# Typologies and Psychological Profiles of Child Sexual Abusers: An Extensive Review

**DOI:** 10.3390/children8050333

**Published:** 2021-04-25

**Authors:** Yeong Yeong Lim, Suzaily Wahab, Jaya Kumar, Fauziah Ibrahim, Mohammad Rahim Kamaluddin

**Affiliations:** 1 Department of Early Childhood Studies, Faculty of Creative Industries, Universiti Tunku Abdul Rahman, Kajang 43000, Malaysia; yylim@utar.edu.my; 2 Centre for Research in Psychology and Human Well-Being, Faculty of Social Sciences and Humanities, Universiti Kebangsaan Malaysia, Bangi 43600, Malaysia; ifauziah@ukm.edu.my; 3 Department of Psychiatry, Faculty of Medicine, Universiti Kebangsaan Malaysia Medical Centre, Cheras, Kuala Lumpur 56000, Malaysia; suzaily@ppukm.ukm.edu.my; 4 Department of Physiology, Faculty of Medicine, Universiti Kebangsaan Malaysia, Cheras, Kuala Lumpur 56000, Malaysia; jayakumar@ukm.edu.my

**Keywords:** child sexual abusers, child sexual offenders, sexual abuse, psychological profile, typology

## Abstract

Child sexual abuse is a public health issue that has been associated with a variety of negative health outcomes. Child sexual abusers constitute a heterogeneous population of individuals. This review lays out an overview of the current understanding of typologies and psychological profiles of child sexual abusers. Typologies of child sexual abusers in general and online child sexual abusers are reviewed to summarise the existing knowledge. Psychological traits including personality traits, cognitive distortion, empathy, and impulsivity are examined to provide a wider perspective of the psycho-criminogenic factors of child sexual abuse. Although past research on child sexual abusers has provided insights into the organisation and classification of different types of child sexual abusers, the classification of these typologies has drawn widespread criticisms. In this review, we discuss the challenges and limitations pertaining to the existing typologies and studies related to the psychological profile of child sexual abusers.

## 1. Introduction

Child sexual abuse is an umbrella term used to describe a wide range of sexual activities that take place between a child and an older person, in which the child does not fully comprehend and is unable to give consent [1]. Numerous terms have been used to illustrate child sexual abuse, including child sexual assault, child sexual victimisation, child exploitation, adverse sexual experience, and unwanted sexual experience [2]. Child sexual abuse is not exclusively committed through physical contact (e.g., rape, molestation, masturbation); it can also take place in the form of nonphysical contact through the internet or manipulation (e.g., production of child pornography, online grooming, exhibitionism) [3,4,5]. Research data derived from 2000 to 2013 indicated that 7 to 12 per cent of children are sexually abused in the United States [6]. Over the years, there was an increase in sexual assault victimisation from 2015 to 2018, which includes children aged 12–17 [7].

Most of the cases involving child sexual abuse are via non-penetrative sexual touch such as fondling and kissing [4,8]. When it comes to the gender of sexual abuse victims, research has been rather conflicting. Even though most research reported more female-oriented victimisation [9,10,11], some studies suggested more male-oriented or no significant difference between gender victimisation [4,12], with the results largely depending on sociodemographic and the nature of sexual abuse studied. Among adult sexual abuse victims, acquaintances and intimate partners are more frequently implicated [13,14,15]. In most child sexual abuse cases, family members are reported to be the common perpetrators. Only one-third of the child sexual abuse was committed by non-family members [4,16]. This estimated incidence derived from data recorded by research and might not be representative of the actual population. Sexual abuses against children are often underreported [5,17,18]. Research indicated different factors such as the locations (urban versus rural), sample size, and methods of collecting data that might influence the estimates of child sexual abuse [10,19,20].

Childhood sexual abuse is proven to have short and long-term negative consequences to the victim. Physical consequences of such abuse include injuries, unintended pregnancy, and genital infections [21]. Those who were sexually abused in childhood are more inclined to develop behavioural and psychological problems such as sleep disturbance, social-related difficulties, eating disorders, self-esteem issues, fear and anxiety, depression, and posttraumatic stress disorder [21,22,23,24,25]. Childhood sexual abuse also increases the risk of future abuse, where the victims are more likely to suffer from domestic violence in their adulthood [26]. From the areas of offence characteristics, those who experience childhood sexual abuse are more likely to commit sexual offences, compared to those without a history of such abuse [23].

Child sexual abusers constitute a heterogeneous population [27]. Although statistics frequently indicated that child sexual abusers are people known to the victims, it does not distinguish the distinct characteristics and motivations of the criminal behaviours exhibited by the perpetrators. Past studies offer various perspectives of knowledge on the characteristics of child sexual abusers, including the classification of multiple subtypes and psychological profiles. Nevertheless, these studies still lack consensus about the classification of child sexual abusers. This review aims to explore and summarise the existing typologies and psychological profiles of child sexual abusers.

The scope of this review focuses on typologies of sexual offenders instead of theories since it provides the key features of the offence behaviours. Typologies merge both theories and practices by classifying criminal behaviours in an organised framework [28]. This review will focus on the factors which are more commonly associated with criminality among child sexual abusers and will not specifically distinguish factors related to psychological disorders. Although psychological disorders such as paedophilia and psychopathy are found to be factors associated with sexual behaviours such as victim selection, levels of involvement in sexual abuse, and response to treatment [29,30,31], these will not be exclusively reviewed.

## 2. Literature Review

### 2.1. Conceptualizing Child Sexual Abusers

Child sexual abuser is an umbrella term used in this current review to cover the broad categories of the sexual abuse perpetrators against children. Based on the articles we have reviewed, the terms referring to child sexual abusers were classified according to the nature of the sexual abuse: contact child sexual abusers and online child sexual abusers (OCSAs). The contact child sexual abuser terms include child molester (CM) [32,33,34,35], child sexual offender (CSO) [36,37,38], contact sex offender against children [32,39,40], child sexual abuser [37,38], child sexual assaulter [36,38,41], sex offender against children [42], perpetrator of abuse [4], and offline child sexual offender [43]. The contact child sexual abusers refer to the sexual abusers who commit their act through physical contact.

There is no single standard term used to refer to OCSAs. Child pornography offender [38,40,43,44,45], child sexual exploitation materials (CSEMs) offender [43], child sexual exploitation materials (CSEMs) user [36,38,41], and online sex offender against children [39,46], internet chat room sex offenders [47], online/internet solicitation offenders [48], indecent images of children (IIOC) offenders [49], and mixed offender (referred to mixed internet/contact CSO) [39] are the terms used to address child sexual abusers who sexually abuse children using online platforms.

### 2.2. Typologies of Child Sexual Abusers

To understand the child sexual abusers, it is necessary to review their existing typologies or classifications. Numerous typologies have been developed over the past 30 years to explain and classify the behaviours of adults who offend against children. Some of these include Massachusetts Treatment Centre: Child Molester Typology–Version 3 (MTC: CM3) [50], child molesters typology [34], child sexual abusers typology by Shevlin et al. [4], internet chat room sex offender typology [47], and online solicitation of children offender’s typology [46].

Knight and Prentky [50] developed MTC: CM3, which is one of the most comprehensive typologies for CM. They classify CM into two main categories (Axis I and Axis II), which evaluate the different characteristics of CM. In Axis I, the CM is classified based on their extent of fixation toward children and measurement of their social competency. In Axis II, CM is classified based on the meaning and levels of contact with children, amount and type of physical injury involved, and their sadistic interest [50]. Based on the two axes, child molesters can be classified into 10 distinct subtypes. The MTC: CM3 was shown to be reliable when replicated in different ethnic groups of CM [35].

Traditional typologies that classify CM into a specific group have difficulties in fitting complex behaviours of CM. Instead of classifying CM into discrete categories, Lanning [34] revised his typologies to include a motivational continuum (situational to preferential). On one side of the continuum, situational child molesters are more likely to be characterised as less intelligent, from a lower socioeconomic status, involved in various unlawful behaviours, consume violent pornographic materials, more impulsive, consider risks rather than needs, likely to make sloppy mistakes, and involved in both spontaneous or planned sexual crimes. On the opposite side of the continuum, the preferential child molesters are more likely to be intelligent, from a higher socioeconomic status, involved in a specific or focused criminal behaviour, consume mostly pornographic materials with specific themes, more compulsive, consider their needs rather than risks, make needy mistakes, driven by fantasy, and engage in ritualistic behaviour patterns [34].

Typologies derived from limited samples of offenders, especially those who have been incarcerated, can potentially misrepresent the actual population. The MTC: CM3 typology that was developed predominantly based on the white sex offender raised doubts over its reliability when applied to other ethnic groups. Furthermore, most of the sexual offences committed are underreported [7]. Due to this reason, participants recruited from correctional or treatment institutions might not entirely reflect the population of child sexual abusers. To address these concerns, Shevlin et al. [4] developed typologies by analysing the information on behaviours of child sexual abusers provided by adult survivors. They classified child sexual abusers into four typologies (labelled intercourse, verbal/low-contact, high sexual contact, and sexual touch). The intercourse type of child sexual abuser has the highest possibility to sexually abuse their victim using penetrative contact in contrast to other sexual abusers who only engage mostly in fondling and kissing [4]. This typology focuses on the characteristic of the crimes to distinguish the different types of offenders. Table 1 summarises the child sexual abusers’ typologies, and the source of the data obtained.

The internet or online sexual crimes against children comprises a range of crimes from distributing and possessing child pornography, production of child pornography, to sexual solicitation [30]. OCSA constitutes different characteristics than general child sexual abusers. OCSA consists of both contact and non-contact sexual abusers even though findings suggest that most OCSAs do not have prior contact sexual offences [47,51]. There has been an increase in child pornography offences and aggressive online solicitation (e.g., involved offline contact) since the year 2000 [52]. Most OCSAs also engage in non-contact exploitative sexual activities such as sending nude photos, taking part in cybersex, and grooming [47]. OCSAs are classified differently than physical child sexual abusers due to the differences in their nature of abuse. With the increasing prevalence of online sexual offences, the different nature of online sexual abuse should be explored further. The easy accessibility of technology and internet connection has changed the landscape of online child sexual exploitation [53]. OCSAs have changed the way they engage their victims with the usage of the dark or deep web, online file sharing and storage, peer-to-peer networking, and streaming services that provide easy access to victims anonymously [54].

Based on OCSA motivation and modus operandi, OCSA typologies are classified according to their severity of offences based on the levels of involvement with the victim. Krone [55] differentiates OCSAs into nine distinguishable types, which are the browser, private fantasy, trawler, nonsecure collector, secure collector, online groomer, physical abuser, producer, and distributor. The browser, private fantasy, and trawler types are usually low in predatory networking involvement. The browser type is those who access CSEMs unintentionally but decided to keep those materials. The private fantasy type uses CSEMs for private use to fulfil their sexual fantasy without the intention of sharing their materials. As for the trawler, they use wide varieties of sexually explicit materials among which CSEMs are just a part of their collection, which has little or no security [55]. As for the non-secure and secure collector type, they engage the use of predatory networks to share, trade, download, or purchase CSEMs [55]. The main difference between these two types of offenders is the level of security measures employed to browse through their materials. Three types of OCSAs—groomer, physical abuser, and producer—can be differentiated from the rest with their direct involvement in the physical abuse of children. The groomer type of OCSAs establishes sexual relationships with children for sexual abuse or desensitizing them to sexual activity. This type of OCSAs may engage in cybersex or physical abuse. In contrast, the physical abuser and producer type would engage in hands-on physical abuse. Although the physical abuser might document their abuse, their motivation is to supplement their sexual craving, unlike producers, whose primary motivation is to provide these documents to other CSEM users [56]. The final type of OCSAs is the distributor, who is involved in possessing CSEM content for distribution and may or may not have a sexual interest in children. The distributor can merely be an opportunistic offender rather than preferential [56].

Briggs et al. [47] proposed a typology for the internet chat room sex offenders that were based on their online sexual behaviours and their engagement in contact offences. According to the typology, there are two types of offenders, contact driven and fantasy driven. The most distinctive characteristic between these two groups is the engagement in offline meetings with the victims. The contact-driven group uses the internet to groom their victims to engage in offline sexual acts. The fantasy-driven group is motivated to achieve their sexual climax through online sexual behaviours such as cybersex and exhibitionism [47]. After the emergence of social networking sites (SNSs) (e.g., Friendster, Myspace, and Facebook) in the early 2000s, chat rooms became obsolete [57]. SNSs is currently reported to be a more popular environment for sexual offending, followed by other online environments such as instant messaging.

DeHart et al. [48] classified different OCSAs by examining the content of their chat logs, emails, and SNS posts. They identified four distinguishable typologies involving online solicitation of children offenders (cybersex offenders, cybersex/scheduler offenders, schedulers, and buyers) [48]. Cybersex and cybersex/schedulers offenders are characterised to be mostly white, expose themselves to the victim, and ask for their victim’s explicit photos. They seldom seek physical interaction or meetups with the victim, but even if they do, they are less likely to turn out to meet their victim (especially the cybersex/schedulers). For the schedulers type of offenders, they are still more likely to be white, but less likely to expose themselves to their victims and more often seeking quick sexual physical interaction. In general, this typology supported the typology proposed by Briggs et al. [47], who found similarities between the different classifications. The scheduler supported the contact-driven offenders, while the cybersex and cybersex/schedulers supported the fantasy-driven offenders [48]. DeHart et al. [48] also identified the fourth type of offenders, the buyers, which is not indicated in previous typologies. This type of offender consisted of more diverse ethnicity, rarely exposed themselves, and more often sought to buy sexual services from victims or their pimps and family members. DeHart et al. [48] distinguished the offenders based on the content of their online communications and did not include other important profiles such as the motivation of the offenders. Table 2 summarises the OCSA typologies, and the source of the data obtained.

### 2.3. Psychological Profile of Child Sexual Abusers

Most of the reviewed typologies focused on the criminological profiles of offenders such as the characteristics of their sexual crime against children, content of evidence, information, and description from victims. Most of the typologies are lacking in distinguishing the psychological profiles of the different offenders. Psychological profiles are important to understand the psycho-criminogenic nature of child sexual abuse. Some of the important psychological profiles include personality traits, cognitive distortion, empathy, and impulsivity. Although there are other psychological profiles that are associated with sexual offending, this review focuses on these few more established profiles that are often linked to criminality due to the vast availability of research.

#### 2.3.1. Personality Traits

Personality is considered a common factor to explain criminal behaviour patterns [58,59,60]. Characteristics of personality or personality traits are descriptions of a person in terms of relatively stable patterns of behaviours, thoughts, and emotions [61]. Although longitudinal research has shown that personality traits do change throughout a lifespan, personality traits are still relatively stable [62]. All studies reviewed in this section are based on findings with convicted and incarcerated child sexual abusers.

In the assessment of child sexual offenders (CSOs), personality traits are considered one of the vital factors since certain personalities might play an important role in child sexual abuse [38]. One of the eminent personality traits often studied in the assessment of CSOs is psychopathy [38]. Psychopathic traits are associated with characteristics such as lack of remorse, lack of empathy, irresponsibility, impulsive, and antisocial behaviours [41]. Findings suggest differences in levels of psychopathy in CSOs (paedophilic and non-paedophilic CSOs) and between sexual offenders and non-offenders. Compared to non-offenders, CSOs are found to exhibit higher levels of psychopathy [38]. Among low-risk CSOs, there was no difference in levels of psychopathy between contact and non-contact CSOs [38], while other studies suggested that non-contact CSOs had lower antisocial tendencies (e.g., personality traits, disregard for rules, lack of remorse, and impulsivity) [43]. Such tendencies are the predictor of sexual contact offences among CSOs [43]. Psychopathy traits could also be one of the factors associated with involvement in situational offences rather than preferential ones. In research comparing paedophilic and non-paedophilic CMs, the non-paedophilic CMs were reported to have higher traits of psychopathy, compared to the paedophilic CMs and non-offenders. The non-paedophilic CMs are more likely to also be involved in various crimes and antisocial acts, and sexual offences against children are one of the various crimes they commit [63].

Apart from psychopathy traits, the five-factor model (FFM) of personality is also studied in describing the profiles of CSOs [32,41]. The personality profiles of sexual offenders differ from non-offenders. Compared to the general public, there are differences in the dimensions of neuroticism, extraversion, and agreeableness among offenders [33]. The neuroticism dimension, which reflects a person’s adjustment and predisposition in experiencing varying negative emotions, was shown to be correlated with child sexual abuse [41]. CSOs were consistently reported to have higher neuroticism than non-offenders [33,38,41], whereas, within offenders, sexual offenders and non-sexual offenders did not differ in the neuroticism trait [33].

Neuroticism is the most consistent personality trait to be associated with both sexual and non-sexual offenders [33,41]. Other traits such as agreeableness, extraversion, and conscientiousness are not as consistent in their relation to CSOs [33,41]. Past findings indicated no differences in the agreeableness dimension of personality among CSOs, non-sexual offenders, and non-offenders. Although a low level of agreeableness is found to be associated with violent offending and criminality in general [64,65], it is not associated with CSOs [33]. This finding might explain the reason why child sexual abusers rarely used violence in their sexual crimes but more often utilised manipulation [48].

Psychological profiles of child sexual abusers are also found to be related to one another. In particular, the FFM personality neuroticism domain is associated with psychopathy [38], cognitive distortion, and other psychological problems [36]. Highly neurotic CSOs tend to experience more negative emotions from daily distress, which offers a possible explanation as to why more psychological problems such as depression, anxiety, anger–hostility, and obsessive–compulsive behaviour are reported among them [36]. High neuroticism is also associated with higher levels of cognitive distortion. The emotional maturity level of the CSO is a contributing factor in shaping such a thinking process [36]. In order to understand the behaviour patterns of child sexual abusers better, there is a need to investigate other psychological profiles that are often associated with these perpetrators further.

#### 2.3.2. Cognitive Distortion

The term cognitive distortion indicates maladaptive beliefs and problematic thinking styles, which include making excuses, blaming, and rationalisation of abusive sexual actions [66]. The child sexual abusers justified their thoughts and actions to rationalise their sexual abuses on children. Many studies have proven the existence of cognitive distortion among sexual offenders [46,67,68]. Compared to non-offenders, child sexual abusers are found to report more cognitive distortions in the areas such as thoughts related to an inability to form and maintaining secure relationships and distorted perceptions of other people’s intentions [68]. Although sexual offenders in general experience more cognitive distortion, when compared within the CSO groups, contact sexual offenders against children reported a higher frequency of cognitive distortion, compared with internet child sexual offenders (non-contact) [39]. Child sexual abusers experience more cognitive distortion in the domain of disconnection/rejection, which is partially related to their fear of rejection. This belief system is likely to influence their relationship since they are unable to maintain secure and satisfactory relationships [68]. This area of cognitive distortion supports their sexual abuse of children. Child contact sexual offenders exhibit more cognitive distortions, compared to the non-contact child internet sexual offenders, especially in the areas of justifying their sexual behaviour, beliefs that children are sexual agents, and in the areas related to power and entitlement over their victims [40]. Similar to the personality traits speculated prior to this, cognitive distortion among contact CSOs was also found to be associated with other psychological profiles such as empathy offenders and distorted attribution of responsibility of their behaviour [37,39].

#### 2.3.3. Empathy

Empathy includes the element of emotion and cognition [69,70]. The emotional perspective of empathy (affective empathy) includes responses such as a feeling of concern for others [70]. The cognitive perspective includes the process such as taking another’s perspective or inferring one’s understanding in others [69,70]. In addition to processing the feelings and perspectives of others, empathy plays an important role in the criminal offences of CSOs [37,39]. Between the groups of offline CSO, child pornographic offender, and mixed offender, the latter has the greatest victim empathy deficit, followed by the offline CSO [46]. Child pornographic offenders are found to have greater victim empathy and a lower level of cognitive distortion. A study comparing the psychological characteristics of different types of child pornographic offenders indicated that offenders who commit contact sexual offences against children are found to report a higher frequency of empathy distortion, compared to the non-contact type of internet child sexual offenders [39]. For instance, those who commit both internet and contact sexual crimes against children are found to report higher personal distress and a higher level of perspective taking, compared to the non-contact internet child sexual offender [39]. Researchers believe that there is a possibility that contact internet child sexual offenders experience cognitive distortions in justifying their offences by distorting their beliefs on the appeals of their sexual behaviours and hence appear to have higher levels of empathy towards their victims. Empathy was also associated with blaming the victim. Low cognitive empathy was related to distorted attribution of more child responsibility. This means that CSOs with low ability to recognise the distress of others are more likely to support the idea that their victims hold responsibility for the sexual crime they have committed [37].

#### 2.3.4. Impulsivity

Impulsivity includes the inability to predict the consequence of one’s actions, along with the thought process related to making impulsive decisions or actions. Impulsivity is also associated with the inability to stop any response that has already been initiated [71]. Factors such as impulsivity increased the risk of having conduct problems throughout childhood [72]. Impulsivity is an important factor in understanding criminal behaviour since it is one of the predictors of reoffending among both sexual offenders and non-sexual offenders [42,73]. Child molesters with high impulsiveness indicated a higher risk of recidivism and having a greater number of victims [73]. Individuals who possess psychopathic qualities, which include impulsiveness, are likely to engage in different criminal behaviours, including sexually abusing children [63]. A study comparing different sex offenders and non-offenders found sex offenders against adults recorded the highest level of impulsivity, compared to CSOs and non-offenders [42]. In addition, contact sexual offenders against children reported the highest cognitive impulsivity scores [39]. Table 3 summarises the psychological profiles of child sexual abusers.

## 3. Discussion

Typologies offer insight into the organisation and classification of different types of child sexual abusers based on their behaviours and motivations. Nevertheless, these typologies have drawn criticisms regarding their classifications. Some of the common challenges in researching this area are the difficulty in operationalising child sexual abusers and the limitation in methodology such as sample selections.

Typologies derived from a collection of data through limited samples of offenders and perpetrators may lead to potential bias or misinterpretation of the actual event. For example, the MTC: CM3 typology was predominantly based on white sex offenders, raising doubts over its validity for use with other ethnic groups [35]. In another research, there was a lack of general agreement about certain offenders, especially the internet/cyber sexual offenders against the children group [47,48]. Such differences may be potentially influenced by advances in technology, especially among OCSAs since their methods of perpetration have changed with easy access to internet platforms and the increasing level of anonymity [54].

Sample selection is limited to the incarcerated or offenders with previous records of child sexual offences. Thus, the samples selected from the aforementioned populations do not fully represent the actual population of child sexual abusers. Furthermore, most of the sexual offences are underreported. In the United States, only an estimated 24.9 per cent of rape or sexual assault cases reported in the year 2018 [7]. Data reviewed in a previous study indicated an estimate of at least 95 per cent of child sexual abuse cases in general were never reported to the authorities [74]. Nonetheless, these estimates should be interpreted with caution since they depend on the source and location of information of the cases that were reported. In short, these statistics indicate that most sexual abusers are off the authority’s radar. As for now, to understand the characteristics and behavioural patterns of child sexual abusers who are not convicted of their crime, researchers were only able to gain insight into this group through information provided by the victims and other recorded information, such as communication logs between the child sexual abusers and their victims [48].

As indicated in the literature reviewed, OCSAs exhibit distinctive profiles from the physical child sexual abusers. Although there are a number of studies investigating the typologies and profiles of OCSAs, OCSAs who sexually abuse their victims for financial gains such as those who produce pornographic materials of children and sextortion are not sufficiently studied [75]. Sexual exploitation and commercialisation involving children (e.g., child prostitution, sex trafficking, sex tourism, and production and consumption of child pornography) are considered as a part of online child sexual abuse [52].

Classification of child sexual abusers is devoid of cultural features in sexual abuses. There are cross-cultural variations in the prevalence of types of child sexual abuse victims from different countries. Studies comparing victims of online child groomers across different countries found that Southeast Asian teenagers are more likely to be targeted, compared to teenagers from Western countries [76]. For contact and penetrative child sexual abusers, Asian countries such as China reported significantly lower prevalence, compared to the international estimates [19]. In sum, there is a need for triangulation in collecting the offenders’ information to limit potential bias, validate the information collected, and better compare the findings.

## 4. Conclusions

Various researchers have employed different processes in classifying child sexual abusers, which can be generally be divided into four, i.e., clinical description, demographic, psychometric profiles, and theory driven. There are no consistent methods used throughout the research, which makes it challenging to compare and synthesise the findings into a comprehensive typology. Additionally, all the research journals reviewed are based on male offenders and abusers. Since the nature of child sexual abuse can be a dynamic process, it is necessary to study child sexual abusers from the perspective of the perpetrators of all genders, victims, and law enforcement records. Moreover, a better risk assessment should be constructed to identify recidivism or the prevalence of sexual abuse against children. Child sexual abusers should be distinguished by their characteristics and motivation of their crimes and their psychological profiles in order to provide a clear understanding of the psycho-criminogenic of their crime. In the future, a more standardised methodology with the inclusion of psychological profile and cultural features in sexual abuses should be developed.

## Figures and Tables

**Table 1:** Summary of child sexual abusers’ typologies and source of data obtained.

Typology	General Classification	Data Obtained in the Study
MTC:CM3 [50]	Axis I Type 0: High fixation, Low social competence Type 1: High fixation, High social competence Type 2: Low fixation, low social competence Type 3: Low fixation, high social competence Axis II Type 1: High contact, Interpersonal Type 2: High contact, Narcissistic Type 3: Low physical Injury, Non-sadistic Type 4: Low physical Injury, Sadistic Type 5: High physical Injury, Non-sadistic Type 6: High physical Injury, Sadistic	Clinical file abstraction (e.g., observation reports, treatment summaries, diagnostic assessment, clinical interviews, etc.) of child molester from treatment centre
Child Molester Typology [34]	Situational Sex Offender Preferential Sex Offender	Case consultation, case study, law enforcement records, and other records (e.g., investigative report, interviews with perpetrator and victim, crime-scene images, etc.)
Typologies of Child Sexual Abuse [4]	Labelled intercourse Verbal/low contact High sexual contact Sexual touch	Treatment information of child sexual abuse victims in treatment centre

**Table 2:** Summary of OCSA typologies and source of data obtained.

Typology	General Classification	Data Obtained in the Study
Typology of Online Child Pornography Offending [55]	Browser, Private Fantasy, Trawler, Nonsecure Collector, Secure Collector, Online Groomer, Physical Abuser, Producer and Distributor	Cases of sexual offences
Chat room sex offender classification [47]	Contact-driven group Fantasy-driven group	Cases of internet initiated sexual offences (e.g., demographic, clinical, and social data)
Typology of online solicitation offenders [48]	Cybersex-only offenders Schedulers Cybersex/schedulers Buyers	Law enforcement case files (e.g., offender chat logs, email threads, and social network posts)

**Table 3:** Summary of child sexual abusers’ psychological profiles.

Psychological Profiles	Types of Child Sexual Abusers	Characteristics	Researcher(s)
Personality traits	General child sexual offenders (no differentiation made for contact, non-contact, and mixed child sexual offenders)	Higher Neuroticism score compared to non-offenders; Lower Extraversion compared to non-offenders. No difference in Neuroticism among non-sexual offenders.	Becerra-García et al. [33]
	Contact child sexual offenders/Child sexual assaulters	Higher Extraversion among 30–50 years old; Lower Conscientiousness for those under 30 years old.	Becerra-García and Egan [32]
		Higher level of psychopathy traits compared to non-offenders; Higher levels of antisocial lifestyle compared to non-offenders; Higher Neuroticism score compared to non-offenders.	Stoll et al. [38]
		Lower Conscientiousness compared to non-offenders; Higher Neuroticism score compared to non-offenders.	Boillat et al. [41]
	Non-contact child sexual offenders/Noncontact child pornography offenders/Child sexual exploitation material users	Lower Conscientiousness compared to non-offenders; Higher Neuroticism score compared to non-offenders.	Boillat et al. [41]
		Lower antisocial tendencies compared to contact CSO.	Babchishin et al. [43]
	Non-paedophilic child Molesters	Higher traits of psychopathy traits compared to paedophilic child molesters and non-offenders.	Strassberg et al. [63]
Cognitive distortion	General child sexual offenders (no differentiation made for contact, non-contact, and mixed child sexual offenders)	Reported more maladaptive schemas compared to non-offenders.	Carvalho and Nobre [68]
	Contact child sexual offenders/Child sexual assaulters	Higher frequency of cognitive distortions compared to internet CSO and mixed child sexual offender.	Elliott et al. [39]
		Exhibit more cognitive distortions compared to the non-contact child internet sexual offenders.	Merdian et al. [40]
	Mixed child sexual offenders (committed both contact and internet child sexual offences)	Exhibit more cognitive distortions compared to the contact only CSO and non-contact child internet sexual offenders.	Merdian et al. [40]
Empathy	Contact child sexual offenders/Child sexual assaulters	Higher frequency of empathy distortion compared to the non-contact type of internet child sexual offenders.	Elliott et al. [39]
	Mixed child sexual offenders (committed both contact and internet child sexual offences)	Highest victim empathy deficit compared to child pornographic offenders and offline CSO.	Babchishin et al. [46]
Impulsivity	General child sexual offenders (no differentiation made for contact, non-contact, and mixed child sexual offenders)	Lower level of impulsivity compared to sexual offenders against adults.	Perley-Robertson et al. [42]
	Contact child sexual offenders/Child sexual assaulters	Higher cognitive impulsivity scores compared to internet child sexual offenders and mixed child sexual offenders.	Elliott et al. [39]

## Data Availability

No new data were created or analyzed in this study. Data sharing is not applicable to this article.

## References

[1] (2017). Responding to Children and Adolescents Who Have Been Sexually Abused: WHO Clinical Guidelines.

[2] Mathews B., Collin-Vézina D. (2019). Child Sexual Abuse: Toward a Conceptual Model and Definition. Trauma, Violence, and Abuse.

[3] Christensen L. (2017). Child Sexual Offenders: The Psychology of Offending. The Psychology of Criminal and Antisocial Behavior: Victim and Offender Perspectives.

[4] Shevlin M., Murphy S., Elklit A., Murphy J., Hyland P. (2018). Typologies of Child Sexual Abuse: An Analysis of Multiple Abuse Acts among a Large Sample of Danish Treatment-Seeking Survivors of Childhood Sexual Abuse. Psychol. Trauma Theory Res. Pract. Policy.

[5] UNICEF East Asia & Pacific (2016). Child Protection in the Digital Age: National Responses to Online Child Sexual Abuse and Exploitation in ASEAN Member States.

[6] Rheingold A.A. (2013). Estimating a Child Sexual Abuse Prevalence Rate for Practitioners: A Review of Child Sexual Abuse Prevalence Studies.

[7] Morgan R.E., Oudekerk B.A. (2019). Criminal Victimization, 2018.

[8] Negriff S., Schneiderman J.U., Smith C., Schreyer J.K., Trickett P.K. (2014). Characterizing the Sexual Abuse Experiences of Young Adolescents. Child Abus. Negl..

[9] Assink M., van der Put C.E., Meeuwsen M.W.C.M., de Jong N.M., Oort F.J., Stams G.J.J.M., Hoeve M. (2019). Risk Factors for Child Sexual Abuse Victimization: A Meta-Analytic Review. Psychol. Bull..

[10] Meinck F., Cluver L.D., Boyes M.E., Loening-Voysey H. (2016). Physical, Emotional and Sexual Adolescent Abuse Victimisation in South Africa: Prevalence, Incidence, Perpetrators and Locations. J. Epidemiol. Community Health.

[11] Pereda N., Abad J., Guilera G. (2016). Lifetime Prevalence and Characteristics of Child Sexual Victimization in a Community Sample of Spanish Adolescents. J. Child Sex. Abus..

[12] Stemple L., Meyer I.H. (2014). The Sexual Victimization of Men in America: New Data Challenge Old Assumptions. Am. J. Public Health.

[13] Black M.C., Basile K.C., Breiding M.J., Smith S.G., Walters M.L., Merrick M.T., Chen J., Stevens M.R. (2011). The National Intimate Partner and Sexual Violence Survey (NISVS): 2010 Summary Report.

[14] Brecklin L.R., Ullman S.E. (2010). The Roles of Victim and Offender Substance Use in Sexual Assault Outcomes. J. Interpers. Violence.

[15] Calkins C., Colombino N., Matsuura T., Jeglic E. (2015). Where Do Sex Crimes Occur? How an Examination of Sex Offense Location Can Inform Policy and Prevention. Int. J. Comp. Appl. Crim. Justice.

[16] Eke A.W., Seto M.C., Williams J. (2011). Examining the Criminal History and Future Offending of Child Pornography Offenders: An Extended Prospective Follow-up Study. Law Hum. Behav..

[17] Zainal Rashid A.A., Kamaluddin M.R., Wahab S., Abdul Aziz D.A., Abdul Latiff Z., Rathakrishnan B. (2018). Vulnerability towards Online Sexual Grooming among Malaysian Children. J. Psikol. Malays..

[18] Nasir N.C.M., Kamaluddin M.R. (2018). Tinjauan Literatur Pemerdagangan Dan Eksploitasi Kanak-Kanak Di Asia Tenggara. J. Pembang. Sos..

[19] Ji K., Finkelhor D., Dunne M. (2013). Child Sexual Abuse in China: A Meta-Analysis of 27 Studies. Child Abus. Negl..

[20] Strand S.J.M., Storey J.E. (2019). Intimate Partner Violence in Urban, Rural, and Remote Areas: An Investigation of Offense Severity and Risk Factors. Violence Against Women.

[21] Tang K., Qu X., Li C., Tan S. (2018). Childhood Sexual Abuse, Risky Sexual Behaviors and Adverse Reproductive Health Outcomes among Chinese College Students. Child Abus. Negl..

[22] Essabar L., Khalqallah A., Benjelloun B.S. (2015). Child Sexual Abuse: Report of 311 Cases with Review of Literature. Pan Afr. Med. J..

[23] Morais H.B., Alexander A.A., Fix R.L., Burkhart B.R. (2018). Childhood Sexual Abuse in Adolescents Adjudicated for Sexual Offenses: Mental Health Consequences and Sexual Offending Behaviors. Sex. Abus..

[24] Noll J.G., Trickett P.K., Long J.D., Negriff S., Susman E.J., Shalev I., Li J.C., Putnam F.W. (2017). Childhood Sexual Abuse and Early Timing of Puberty. J. Adolesc. Health.

[25] Okeafor C.U., Okeafor I.N., Tobin-West C.I. (2018). Relationship Between Sexual Abuse in Childhood and the Occurrence of Mental Illness in Adulthood: A Matched Case–Control Study in Nigeria. Sex. Abus. J. Res. Treat..

[26] Barrios Y.V., Gelaye B., Zhong Q., Nicolaidis C., Rondon M.B., Garcia P.J., Sanchez P.A.M., Sanchez S.E., Williams M.A. (2015). Association of Childhood Physical and Sexual Abuse with Intimate Partner Violence, Poor General Health and Depressive Symptoms among Pregnant Women. PLoS ONE.

[27] Levine J.A., Dandamudi K. (2016). Prevention of Child Sexual Abuse by Targeting Pre-Offenders Before First Offense. J. Child Sex. Abuse.

[28] Helfgott J.B. (2013). Criminal Typologies. Criminal Psychology.

[29] Turner D., Rettenberger M., Lohmann L., Eher R., Briken P. (2014). Pedophilic Sexual Interests and Psychopathy in Child Sexual Abusers Working with Children. Child Abus. Negl..

[30] Seto M. (2015). Internet-Facilitated Sexual Offending.

[31] Seto M.C., Harris G.T., Lalumière M.L. (2016). Psychopathy and Sexual Offending. Personality and Clinical Psychology Series. The Clinical and Forensic Assessment of Psychopathy: A practitioner’s Guide.

[32] Becerra-García J.A., Egan V. (2014). A Cross-Sectional Study of the Relationships between Age and Personality in Sex Offenders against Children. Sexologies.

[33] Becerra-García J.A., García-León A., Muela-Martínez J.A., Egan V. (2013). A Controlled Study of the Big Five Personality Dimensions in Sex Offenders, Non-Sex Offenders and Non-Offenders: Relationship with Offending Behaviour and Childhood Abuse. J. Forensic Psychiatry Psychol..

[34] Lanning K.V. (2010). Child Molesters: A Behavioral Analysis For Professionals Investigating the Sexual Exploitation of Children.

[35] Schaaf S., Jeglic E.L., Calkins C., Raymaekers L., Leguizamo A. (2016). Examining Ethno-Racial Related Differences in Child Molester Typology: An MTC:CM3 Approach. J. Interpers. Violence.

[36] Boillat C., Deuring G., Pflueger M.O., Graf M., Rosburg T. (2017). Neuroticism in Child Sex Offenders and Its Association with Sexual Dysfunctions, Cognitive Distortions, and Psychological Complaints. Int. J. Law Psychiatry.

[37] Hempel I.S., Buck N.M.L., van Vugt E.S., van Marle H.J.C. (2015). Interpreting Child Sexual Abuse: Empathy and Offense-Supportive Cognitions among Child Sex Offenders. J. Child Sex. Abus..

[38] Stoll C.B., Boillat C., Pflueger M.O., Graf M., Rosburg T. (2019). Psychopathy, Neuroticism, and Abusive Behavior in Low Risk Child Sex Offenders. J. Child Sex. Abus..

[39] Elliott I.A., Beech A.R., Mandeville-Norden R. (2013). The Psychological Profiles of Internet, Contact, and Mixed Internet/Contact Sex Offenders. Sex. Abus. J. Res. Treat..

[40] Merdian H.L., Curtis C., Thakker J., Wilson N., Boer D.P. (2014). The Endorsement of Cognitive Distortions: Comparing Child Pornography Offenders and Contact Sex Offenders. Psychol. Crime Law.

[41] Boillat C., Schwab N., Stutz M., Pflueger M.O., Graf M., Rosburg T. (2017). Neuroticism as a Risk Factor for Child Abuse in Victims of Childhood Sexual Abuse. Child Abus. Negl..

[42] Perley-Robertson B., Maaike Helmus L., Derkzen D., Serin R.C. (2016). Do Sex Offenders against Adults, Sex Offenders against Children, and Non-Sex Offenders Differ in Impulsivity. Sex. Offender Treat..

[43] Babchishin K.M., Merdian H.L., Bartels R.M., Perkins D. (2018). Child Sexual Exploitation Materials Offenders: A Review. European Psychologist.

[44] Magaletta P.R., Faust E., Bickart W., McLearen A.M. (2014). Exploring Clinical and Personality Characteristics of Adult Male Internet-Only Child Pornography Offenders. Int. J. Offender Ther. Comp. Criminol..

[45] Price M., Lambie I., Krynen A.M. (2015). New Zealand Adult Internet Child Pornography Offenders. J. Crim. Psychol..

[46] Babchishin K.M., Hanson R.K., VanZuylen H. (2015). Online Child Pornography Offenders Are Different: A Meta-Analysis of the Characteristics of Online and Offline Sex Offenders Against Children. Arch. Sex. Behav..

[47] Briggs P., Simon W.T., Simonsen S. (2011). An Exploratory Study of Internet-Initiated Sexual Offenses and the Chat Room Sex Offender: Has the Internet Enabled a New Typology of Sex Offender?. Sex. Abus. J. Res. Treat..

[48] DeHart D., Dwyer G., Seto M.C., Moran R., Letourneau E., Schwarz-Watts D. (2017). Internet Sexual Solicitation of Children: A Proposed Typology of Offenders Based on Their Chats, e-Mails, and Social Network Posts. J. Sex. Aggress..

[49] Long M.L., Alison L.A., McManus M.A. (2013). Child Pornography and Likelihood of Contact Abuse: A Comparison Between Contact Child Sexual Offenders and Noncontact Offenders. Sex. Abus. J. Res. Treat..

[50] Knight R.A., Prentky R.A. (1990). Classifying Sexual Offenders. Handbook of Sexual Assault.

[51] Wolak J., Finkelhor D., Mitchell K. (2011). Child Pornography Possessors: Trends in Offender and Case Characteristics. Sex. Abus. J. Res. Treat..

[52] Quayle E. (2016). Researching Online Child Sexual Exploitation and Abuse: Are There Links between Online and Offline Vulnerabilities?.

[53] Westlake B.G. (2020). The Past, Present, and Future of Online Child Sexual Exploitation: Summarizing the Evolution of Production, Distribution, and Detection. The Palgrave Handbook of International Cybercrime and Cyberdeviance.

[54] Henry N., Flynn A. (2019). Image-Based Sexual Abuse: Online Distribution Channels and Illicit Communities of Support. Violence Women.

[55] Krone T. (2004). A Typology of Online Child Pornography Offending.

[56] Bang B., Baker P.L., Carpinteri A., van Hasselt V.B. (2014). Commercial Sexual Exploitation of Children.

[57] Wagstaff K. (2012). The Good Ol’ Days of AOL Chat Rooms.

[58] Kamaluddin M.R., Syariani N., Ayu G. (2013). A Validity Study of Malay Translated Zuckerman-Kuhlman Personality Questionnaire Cross-Cultural 50 Items (ZKPQ-50-CC). Health Environ. J..

[59] Kamaluddin M.R., Shariff N.S.M., Othman A., Ismail K.H., Mat Saat G.A. (2015). Linking Psychological Traits with Criminal Behaviour: A Review. Asean J. Psychiatry.

[60] Kamaluddin M.R., Shariff N.S.M., Nur-Farliza S., Othman A., Ismail K., Mat Saat G.A. (2014). Personality Traits as Predictors of Low-Self Control, Aggression and Self-Serving Cognitive Disorder: A Study Among Malaysian Male Murderers. J. Psikol. Malays..

[61] McCrae R.R., Costa P.T. (2003). Personality in Adulthood.

[62] Bleidorn W., Hopwood C.J., Lucas R.E. (2018). Life Events and Personality Trait Change. J. Personal..

[63] Strassberg D.S., Eastvold A., Wilson Kenney J., Suchy Y. (2012). Psychopathy among Pedophilic and Nonpedophilic Child Molesters. Child Abus. Negl..

[64] Van Dam A. (2018). Less than 1% of Rapes Lead to Felony Convictions. At Least 89% of Victims Face Emotional and Physical Consequences. The Washington Post.

[65] Walters G.D. (2018). Personality and Crime: Mediating the Agreeableness–Offending and Conscientiousness–Offending Relationships with Proactive and Reactive Criminal Thinking. Personal. Individ. Differ..

[66] Ward T., Casey A. (2010). Extending the Mind into the World: A New Theory of Cognitive Distortions in Sex Offenders. Aggress. Violent Behav..

[67] Beech A.R., Bartels R.M., Dixon L. (2013). Assessment and Treatment of Distorted Schemas in Sexual Offenders. Trauma Violence Abus..

[68] Carvalho J., Nobre P.J. (2014). Early Maladaptive Schemas in Convicted Sexual Offenders: Preliminary Findings. Int. J. Law Psychiatry.

[69] Cuff B.M.P., Brown S.J., Taylor L., Howat D.J. (2016). Empathy: A Review of the Concept. Emotion Review.

[70] Pulos S., Elison J., Lennon R. (2004). The Hierarchical Structure of The Interpersonal Reactivity Index. Soc. Behav. Personal. Int. J..

[71] Krasowska A., Wojnar M. (2013). Impulsivity in Sexual Offenders—New Ideas or Back to Basics?. Psychiatr. Polska.

[72] Pardini D.A., Byrd A.L., Hawes S.W., Docherty M. (2018). Unique Dispositional Precursors to Early-Onset Conduct Problems and Criminal Offending in Adulthood. J. Am. Acad. Child Adolesc. Psychiatry.

[73] Baltieri D.A., Boer D.P. (2015). Two Clusters of Child Molesters Based on Impulsiveness. Rev. Bras. Psiquiatr..

[74] Martin E.K., Silverstone P.H. (2013). How Much Child Sexual Abuse Is “below the Surface,” and Can We Help Adults Identify It Early?. Front. Psychiatry.

[75] Wager N., Armitage P.R., Christmann K., Gallagher B., Ioannou M., Parkinson S., Reeves C., Rogerson M., Synnott J. (2018). Rapid Evidence Assessment: Quantifying Online Facilitated Child Sexual Abuse: Report for the Independent Inquiry into Child Sexual Abuse.

[76] Wachs S., Jiskrova G.K., Vazsonyi A.T., Wolf K.D., Junger M. (2016). A Cross-National Study of Direct and Indirect Effects of Cyberbullying on Cybergrooming Victimization via Self-Esteem. Psicol. Educ..

